# Altered CSF Proteomic Profiling of Paediatric Acute Lymphocytic Leukemia Patients with CNS Infiltration

**DOI:** 10.1155/2019/3283629

**Published:** 2019-05-02

**Authors:** Fei Mo, Xuelei Ma, Xiaobei Liu, Ruofan Zhou, Yunuo Zhao, Hui Zhou

**Affiliations:** Department of Biotherapy, Cancer Center, State Key Laboratory of Biotherapy, West China Hospital, Sichuan University, China

## Abstract

**Background:**

For childhood acute lymphocytic leukemia (ALL), central nervous system leukemia (CNSL) is still the main reason of treatment failure. Changes of cerebrospinal fluid (CSF) proteome are deemed to occur after intrathecal chemotherapy.

**Objective:**

To find critical CSF biomarkers, which could be utilized to increase diagnostic and prognostic accuracy of CNSL.

**Methods:**

We performed proteomic profiling of CSF before and after the treatment of six sporadic paediatric patients diagnosed as ALL with central nervous system (CNS) involvement. CSF samples were properly processed and analyzed through the use of label-free liquid chromatography-tandem mass spectrometry (LC-MS/MS).

**Results:**

Among identified 428 unique proteins in all CSF samples, we quantified 10 altered proteins with diverse biological functions after induction chemotherapy.

**Conclusions:**

The levels of those 10 proteins change during the treatment of CNSL. Some of the proteins are likely to play a vital biological role as biomarkers for the development of ALL. In addition, our results indicated the feasible and reproducible utility of CSF for diagnosis and prognosis of patients with CNSL.

## 1. Introduction

ALL is the most frequent malignancy in children and the peak incidence pertains to 1–5 years age cohort [1]. Recently, a study showed that during 2010–2014 in 14 countries, the lowest rate of 5-year survival of paediatric patients with ALL was of Ecuadorians (49.80%), and the highest was of Finns (95.20%), the range of which was elevated 10% or more compared with that in 1995 [2]. Paediatric ALL has been considered to be potentially curable as a result of the rapidly improved cytogenetic, molecular, and immunophenotyping stratification of leukemic blasts and risk-directed treatment [3–5]. However, CNS relapse still occurs in 3–8% of the children with ALL and it is a major factor causing death related to cancer of children over 5. Moreover, patients with CNSL are apt to have a relatively poor outcome compared with CNS-negative patients [6]. Therefore, in patients with CNSL, a more sensitive and noninvasive diagnosis method and new biomarkers reflecting treatment response and prognosis are in urgent need [7].

To date, CSF proteomics has rapidly developed and become a new method of diagnosis, treatment, and prognosis of a wide range of diseases, especially nervous system-relative disorders [8], such as Alzheimer, disseminated sclerosis Parkinson, chronic nervous headache, acute brain injury, and mental disorders [9–12]. Increased evidence showed that the proteomic analysis of CSF could also provide candidate protein biomarkers for brain tumours [13], especially glioma [14]. Unfortunately, few researches aimed at finding the CSF proteome change of hematological malignancies [15]. Therefore, we undertook a research focusing on the quantitative proteomics of CSF in patients with nasal-type of extranodal natural killer cell/T-cell lymphoma (NKTCL) [16]. In the present study, we performed a high-throughput quantitative CSF proteomic analysis of patients with CNSL before and after conventional treatment by label-free LC-MS/MS. We found that the levels of ten proteins with different locations and functions significantly changed during the induction chemotherapy for ALL. These proteins are associated with inflammatory processes and tumour development in varying degrees. Thus, this work may provide useful information for molecular mechanisms of ALL development, as well as predicting clinical prognosis of patients with ALL on high risk of CNS infiltration.

## 2. Materials and Methods

### 2.1. Participants and CSF Collection

Eligible patients, aged 1-11 years, had confirmed B-lineage ALL with CNS involvement when the white blood cells were 1-5/*μ*L with detectable leukemic blasts in CSF. These patients received additional intrathecal chemotherapy (ITC) involving methotrexate, cytarabine, and dexamethasone weekly during induction (comprised prednisolone, daunorubicin, vincristine, and pegaspargase according to the protocols of CCCG-ALL-2015). CSF samples were obtained by lumbar puncture from subjects at Pediatric Hematology of West China Second University Hospital, Sichuan University. The CSF samples collected before the treatment and after achieving a complete response were divided into two groups: PRE-CSF and POST-CSF, respectively. After standard checks in laboratory, all CSF samples were spun at 3000 rpm under the temperature of 4°C for 10 min to remove any cellular debris and were then stored in aliquots under the temperature of −80°C after 2 h of harvesting. The Medical Ethics Committee belonging to Sichuan University's West China Hospital confirms the study. Also, the informed consents had been signed by all these patients before they hospitalized.

### 2.2. CSF Sample Preparation

Aliquots of CSF were dissolved at room temperature. Also, the dilution of a fixed amount of CSF (30 *μ*L) was lysed in RIPA buffer (150mm NaCl, 50 mm Tris-HCl (pH 7.61), NP-40, 1% deoxycholic acid) with phosphatase and protease inhibitors for 20 min on ice. After 10 min's centrifugation at 13000 x g under the temperature of 4°C, determination of the supernatant protein concentrations was finished by BCA assay. The buffer containing 100 mM NH_4_HCO_3_ was added to obtain the 50 mM NH_4_HCO_3_'s final protein concentration, making an alkaline environment for the trypsin digestion. Then the samples were reduced with a final concentration of 5 mM DL-Dithiothreitol (DTT) for 1 h under the temperature of 56°C. For the purpose of alkylating the cysteines, 55 mM iodoacetamide (IAA) was added to reach the concentration of 15 mM, which then reacted in darkness under room temperature for 30 min. Then 30 mM L-cysteine was added to block redundant IAA, and digestion of protein samples was finished with trypsin (Promega) at a ratio of 50:1 (protein to trypsin) under the temperature of 37°C overnight under pH 8.0. Finally, the samples were heated to the temperature of 95°C for 10 min to stop the reaction. Before LC−MS/MS analysis, desalination of the peptides was finished by C18 ZipTip (Millopore, ZTC18S096).

### 2.3. Liquid Chromatography-Tandem Mass Spectrum Analysis

During the LC-MS/MS analysis process, the same method of our previous report was adopted [16]. In brief, all these lyophilized peptide digests were resuspended in buffer A (2.0% ACN, 0.1% FA), after which LC-MS/MS analysis was conducted in triplicate by nanoflow EASY-nLC system, coupled online to Q-Exactive quadrupole-orbitrap mass spectrometer (Thermo Fisher Scientific) with a nanoelectrospray ionization source. The determination of run orders for all the samples was made in a random manner. A 100 *μ*m x 2 cm trap column (200A, 5 *μ*m; Michrom Bioresources) was used to perform separations, and then the trap column was switched with an analytical column of 75 *μ*m x 12 cm (200A, 5 *μ*m; Michrom Bioresources).

A 3 min column washing was conducted with mobile phase A (2.0% ACN, 0.1% FA). The 60-min liquid-chromatography (LC) gradient was used with a rising percentage of buffer B (95.0% ACN, 0.1% FA) from initially 4% to 90% for peptide elution purposes (flow rate: 300 nL/min). Data were collected by positive ion mode. MS spectra selection scan range was m/z 375 – 1800 (resolution: 70,000, m/z: 200). The operation of the LTQ-Orbitrap was finished in data-dependent acquisition mode. The value of automatic gain control (AGC) was set at 3e6. The top 15 most intense parent ions (number: 20) were selected and the fragmentation was performed in HCD collision cell (the set mass resolution: 35000). For the purpose of preventing repetitive selection of peptide, a 30 s' dynamic exclusion was applied. Collection of the raw files was acquired using Q-Exactive, after which they were analyzed by Maxquant v1.3. The searching was accomplished according to Swiss-Prot human database. To ensure the confidence in these listed proteins, identification of peptides was made with at least 1 unique peptide with a peptide false discovery rate <1% on the basis of Peptide Prophet Algorithm in Elucidator. Five missed trypsin cleavages were allowed; cysteine carbamido methylation was set as a fixed modification in this search.

### 2.4. Proteomics Enrichment Analysis

After filtering the data set, enrichment analysis of the CSF proteome was taken in Gene Ontology (GO). Then, the identified proteins' Kyoto Encyclopedia of Genes and Genomes (KEGG) pathways were carried out using David 6.8 (https://david-d.ncifcrf.gov/). Significantly altered-protein expression profiles were present by heatmap in Mev software. Finally, we took visualized known STRING actions (https://string-db.org/) for significantly changed CSF proteins [17].

### 2.5. Western Blot

CSF samples were diluted in 5x SDS-PAGE sample loading buffer (Beyotime) after determining the protein concentration and heated at 95°C for 10 minutes. Fifty micrograms of total proteins from each sample were loaded into each well, then separated on 12.5% SDS-PAGE gel (Bio-Rad) and transferred to the polyvinylidene difluoride membrane (Millipore). After incubation with blocking solution (containing 5% milk) for 120 min at room temperature, membranes were incubated with the following primary antibodies overnight at 4°C: rabbit-anti-HRG (Abcam), rabbit-anti-SPARC (Abcam). Then, membranes were washed three times and incubated with horseradish peroxidase-conjugated secondary antibody (Thermo Fisher Scientific) at 37°C for 1 h. Chemiluminescent visualization (Clinx Science Instruments) was used to visualize the signals. Western blot data were normalized by Image J software.

### 2.6. Statistical Analysis

Multiple comparisons of PRE-CSF and POST-CSF samples were performed using paired t-test. The analysis of protein profiles was implemented for candidate proteins using univariate analysis. We filtered our list of significant altered proteins at p value <0.05. P values were calculated in accordance with Mann–Whitney U test for nonparametric data, or the two-tailed t-test for parametric data. The data were shown as means ± standard deviations (SD).

## 3. Results

### 3.1. Global CSF Protein Analysis

**Figure 1:**
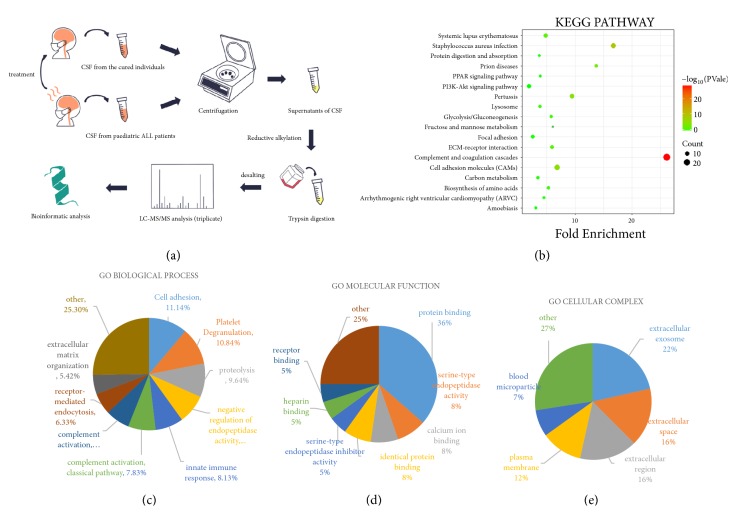
The comprehensive proteomics analysis of CSF with CNSL. (a) The experimental work flow. The process of sample preparation, data acquisition, and analysis is shown in the diagram. ALL, leukeamia. (b) In-depth KEGG-pathway analysis of all identified proteins in PRE-CSF samples. (c), (d), and (e) Gene Ontology (GO) analysis of all identified proteins expressed in PRE-CSF samples. All identified proteins were put into the three GO domains (BP, MF, and CC). The names and percentages of term are located next to their position on the charts.

Ultimately, six eligible patients were included in our study. The demonstration of our experimental workflow was presented in Figure 1(a). To investigate the proteomic profile of CSF in patients with CNSL, we collected PRE- and POST-CSF samples and used the same volume of samples for subsequent laboratory tests and analysis. Within these pooled CSF samples, an average of 428 unique proteins of all PRE-CSF and POST-CSF samples was identified by measuring peptides signal intensity for protein abundance (Supplementary Table S1). During the treatment, the following pathway was revealed activated by depth KEGG-pathway analysis: complement and coagulation cascades, related to the usage of antitumor drugs during induction therapy to ALL [18] (Figure 1(b)). Next, we grouped those proteins according to annotations in Gene Ontology (GO) domains, including ‘Biological Process (BP)', ‘Molecular Function (MF)', and ‘Cellular Complex (CC)', to determine the constitutions of the CSF proteome. Then, the percentage representation of each GO subcategory domain was demonstrated with all expressed proteins. There was no substantial difference among the three subcategories proportions between pretreated and posttreated groups of patients with ALL (Supplementary Figure S1). The analysis in BP ontology showed significant protein enrichment in the cell adhesion, platelet degranulation, and proteolysis (Figure 1(c)). MF analysis indicated that most of the identified proteins were noted as protein binding and calcium ion binding as well as serine−type endopeptidase inhibitor activity (Figure 1(d)). Analysis of CC ontology mainly pointed towards extracellular exosome, extracellular space, and extracellular region (Figure 1(e)).

### 3.2. Label-Free Quantification of Relevant Protein Abundance

**Figure 2:**
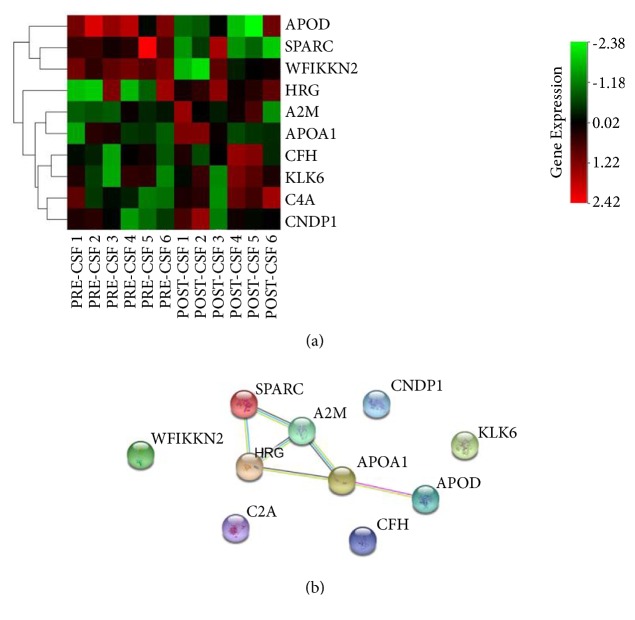
Quantification of changed CSF proteins during the treatment of CNSL. (a) Heatmap analysis of deproteins in PRE-CSF and POST-CSF group: unsupervised clustering analysis showing expression profiles of DE proteins with Euclidean Distance of proteins. (b) The altered-protein interactions generated by STRING analysis. Required confidence (score): medium confidence (0.400). Edge width reflects the strength of the STRING action score and the edge length is arbitrary.

Aiming at identifying differentially expressed proteins in these PRE-CSF and POST-CSF samples, we performed a strict t-test difference criteria (Method section) and several statistically significant proteins were identified (Table 1). The expression of all these proteins was presented in Figure 2(a). Our label-free quantification demonstrated that the following seven proteins were upregulated: histidine rich glycoprotein (HRG), kallikrein related peptidase 6 (KLK6), carnosine dipeptidase 1 (CNDP1), alpha-2-macroglobulin (A2M), complement factor H (CFH), complement C4A (C4A), and apolipoprotein A1 (APOA1). On the contrary, WAP, follistatin/kazal, immunoglobulin, kunitz and netrin domain including 2 (WFIKKN2), secreted protein acidic as well as cysteine rich (SPARC), apolipoprotein D (APOD) were significantly downregulated. In addition, we used western blotting technique to validate the LC-MS/MS results, demonstrated in Supplementary Figure S2. HRG and SPARC were selected for the confirmation of the low intensity and potential applied value as the biomarkers of ALL. Consistent with our LC-MS/MS results, the content of SPARC was obviously decreased (p=0.044) and HRG had the trend of increasing (p=0.072) after treatment of ALL. The protein interaction among these 10 proteins is showed in Figure 2(b). Therefore, these proteins are possibly related to ALL development directly or indirectly, and their changes during the treatment might be used as an indication of the better therapeutic effects in CNS.

## 4. Discussion

In this research, the label-free LC−MS/MS was adopted for constructing overall CSF proteomic profiles of pediatric CNSL samples. Strong concordance could be seen in the proportion of identified proteins assigned to BP, MF, and CC of GO domains between PRE and POST-CSF samples from our data, demonstrating that the overall composition of CSF proteome is relatively stable [16]. In addition, the different points in relative levels of CSF proteins were detected in CNSL patients during induction. As subclinical CNS manifestation is in most patients, CNS-directed therapy is critical for increasing leukemia-free survival rates, even in nondetectable CNSL pediatric patients [19]. Intrathecal chemotherapy (ITC) with methotrexate or cytarabine is commonly used to prevent or treat CNSL. However, various adverse events are likely to occur, including severe gastrointestinal, hepatic and neurological toxicities, leukoencephalopathy, and polyradiculopathy [20, 21]. Therefore, improving therapy efficacy and reducing toxicity are the current focus of ALL management [22]. CSF is proximate to CNS tissue microenvironment and has relative simplex protein contents; thus, it is preferentially considered as an ideal biomarker source for neurological disorders [23]. By analyzing the changes of expressed proteins during ITC in patients with CNSL, we could find new markers to develop alternative CNSL-related diagnosis, prognosis, and treatment strategies.

In this work, we found the levels of three proteins (SPARC, WFIKKN2, and APOD) decreased during CNSL treatment. SPARC is a 32 kDa secreted glycoprotein that regulates interaction of cell-extracellular matrix proteins to promote cell adhesion and migration [24]. It is also reported to be important for tissue remodelling, angiogenesis, and tumourigenesis through activation of growth factors and matrix metalloproteinases [25–28]. Increased expression of SPARC has been found to play a protumorigenic and prometastatic role to many malignant tumours, such as brain, lung, breast, pancreas, skin, and kidney [29]. Another study showed that the downregulated or absent expression of SPARC is related to high-speed progression of human colon cancer [30]. Thus, SPARC may have complex function in the development of human solid tumours. However, there are few studies discussing the role of SPARC in the formation of hematological malignances. Recently, accumulating evidence suggested that SPARC overexpression is associated with poor outcome in acute myeloid leukemia (AML) patients and promotes the growth of aggressive leukemia cell in murine models. In addition, knockdown of SPARC could inhibit AML cell proliferation through inducing cell cycle arrest at Phase G1/G0 and inhibit active p53-induced apoptosis through raising the levels of p53, caspase-9, caspase-3, and Fas [24, 31]. As a marker of poor prognosis and a potential therapeutic target of AML, a similar mechanism may be helpful for inhibiting ALL development, and the decreased level of SPARC may account for a better prognosis of CNSL, since the levels of SPARC significantly declined in subjects when they CR was achieved in our study. WFIKKN2 is a large extracellular multidomain protein, containing a WAP, an immunoglobulin, a follistatin domain (binds mature growth factor), an NTR domain, and two Kunitz-type protease inhibitor domains [32]. It is known to positively activate PI3K pathway, which could regulate cancer cell growth and progression [33]. Moreover, it can promote the combination of TGF*β* family members to the receptor to regulate the balanced relation between the activation of non-Smad and Smad pathways of TGF*β* [34]. As a 33-kDa glycoprotein component of high-density lipoprotein in plasma, Apo D is observably expressed by glial cells in brain [35]. It is essential for the development and repair of nervous system as an important regulator of lipid trafficking and conferring protection from cell oxidative stress [36, 37]. Apo D could also act as a nonspecific stress protein leading to cell growth arrest, and it is upregulated under several pathological situations such as cancers and neurodegenerative diseases [38, 39].

Increased levels of C4A and CFH were involved in complement and coagulation cascades pathway [40]. Furthermore, CFH inhibits endothelial cell migration and angiogenesis by indirectly regulating angiogenic effectors of complement components, including C3a and C5a, leading to lower vascular endothelial growth factor (VEGF) expression and transformation of growth factor-*β* (TGF-*β*) [41–43]. The upregulated expression of CFH might be beneficial to confront VEGF produced by ALL cells, as VEGF has been proved to promote leukemia cell infiltration into CNS [36]. As a 720-kDa glycoprotein, A2M is mainly produced by liver Kupffer cells. A2M is also involved in inflammatory reaction as a proteinase inhibitor and carrier for interleukin-6 (IL-6) [37]. It has been shown to decrease in some pathological conditions such as hematologic malignancy and advanced prostate cancer [44, 45]. It was demonstrated previously that the interaction of it with the lipoprotein receptor-related protein-1(LRP1) of low-density led to an inhibition of tumour cell proliferation, migration, and invasion in astrocytoma malignancy [46]. HRG is an 75 kDa a2-glycoprotein produced by liver, which keeps high levels in plasma and acts as an acute phase reactant [47]. HRG is involved in numerous biologic processes, such as cell adhesion and proliferation, angiogenesis as well as coagulation and platelet activation [48]. It is also reported that HRG inhibits tumour growth and vascularization, remodels transition from epithelial to mesenchymal, and regulates the formation of protumorigenic microenvironment by skewing tumour-associated macrophages polarization from the M2-like towards M1-like phenotype [49–51]. Besides, HRG promotes antitumor immune responses and vessel normalization to enhance chemotherapy. KLK6, a member of the secreted serine protease family of protein-cleaving enzymes, is also defined as an epigenetically regulated tumour suppressor [52]. KLK6 expression resulted in significant downregulation of vimentin, a critical marker of epithelial-to-mesenchymal transition of tumour cells [53]. APOA1, the major protein component of high-density lipoproteins (HDL) particles in plasma, suppressed neutrophil recruitment in inflammation to avoid excessive inflammatory response [54]. Furthermore, it is a potential antiangiogenic factor to suppress tumour progression by targeting c-Src/ERK signaling pathways [55, 56]. Carnosinase 1(CNDP1), primarily synthesized in brain, secreted into CSF, and finally secreted into the blood, is likely to be important in the maintenance and bioavailability of carnosine, a drug for tumours. It could be verified that CNDP1 has antiproliferative properties on different malignant cells for its therapeutic potential against tumours including glioblastoma and metastatic prostate cancer. Its deficiency has been related to many kinds of neurologic deficits and cancer cachexia [57–59]. Several statistically altered proteins have been previously proved to play a role of regulation in malignant cells or microenvironment of cancers, which might be related to the molecular mechanisms of ALL development and prognosis of patients with CNSL.

Collectively, we constructed CSF's label-free LC−MS/MS proteomic profiles in patients suffering from CNSL, which helps us identify valuable biomarkers to understand the development of ALL with CNS infiltration. It is noteworthy that those altered proteins should be furtherly investigated as predictive markers of ALL patients with CNS infiltration, some of which may have a prospect of becoming the new therapeutic targets in CNSL. However, this study has some limitations. For example, the size of samples is small since only 6 subjects are covered, and the period of observation is relatively short. Therefore, we will enlarge the sample size and prolong the observation time in our next study, hoping to further verify the roles of those altered proteins in paediatric patients of ALL with CNS infiltration.

## Figures and Tables

**Table 1:** Statistically significant increased or decreased proteins in ALL samples during induction with P<0.05.

Protein	Gene name	Protein ID	Change trend	P value
complement C4A (Rodgers blood group)	C4A	P0C0L4	↑	0.011
Histidine rich glycoprotein	HRG	P04196	↑	0.016
apolipoprotein A1	APOA1	P02647	↑	0.025
kallikrein related peptidase 6	KLK6	Q92876	↑	0.025
carnosine dipeptidase 1	CNDP1	Q96KN2	↑	0.026
complement factor H	CFH	P08603	↑	0.029
alpha-2-macroglobulin	A2M	P01023	↑	0.05

apolipoprotein D	APOD	P05090	↓	0.018
WAP, follistatin/kazal, immunoglobulin, kunitz and netrin domain containing 2	WFIKKN2	Q8TEU8	↓	0.024
secreted protein acidic and cysteine rich	SPARC	P09486	↓	0.037

## Data Availability

The data from LC-MS/MS analysis used to support the findings of this study are included within the article.

## Abbreviations

A2M: Alpha-2-macroglobulin
ACN: Acetonitrile
AGC: Automatic gain control
ALL: Acute lymphocytic leuk(a)emia
AML: Acute myeloid leuk(a)emia
APOA1: Apolipoprotein A1
APOD: Apolipoprotein D
BP: Biological Process
C4A: Complement C4A
CC: Cellular Complex
CFH: Complement factor H
CID: Collision induced dissociation
CNDP1: Carnosine dipeptidase 1
CNS: Central nervous system
CNSL: Central nervous system leuk(a)emia
CSF: Cerebrospinal fluid
DTT: DL-Dithiothreitol
FA: Formic acid
GO: Gene Ontology
HCD: Higher energy Collision induced Dissociation
HDL: High-density lipoproteins
HRG: Histidine rich glycoprotein
IAA: Iodoacetamide
IL-6: Interleukin-6
IT: Intrathecal therapy
ITC: Intrathecal chemotherapy
KEGG: Kyoto Encyclopedia of Genes and Genomes
KLK6: Kallikrein related peptidase 6
LC: Liquid-chromatography
LC-MS/MS: Liquid chromatography-tandem mass spectrometry
LRP1: Lipoprotein receptor-related protein-1
LTQ: Linear trap
MF: Molecular Function
NKTCL: Natural killer cell/T-cell lymphoma
POST-CSF: After achieving a complete response
PRE-CSF: Before the treatment
SD: Standard deviations
SPARC: Secreted protein acidic and cysteine rich
TGF-*β*: Transforming growth factor-*β*
VEGF: Vascular endothelial growth factor
WAP: Whey acidic protein
WFIKKN2: WAP, follistatin/kazal, immunoglobulin, kunitz and netrin domain containing 2.

## Supplementary Materials

Supplementary Materials Supplementary Figure S1: Gene Ontology (GO) analysis of all identified proteins expressed in POST-CSF samples. Supplementary Figure S2: validation of SPARC and HRG protein peaks by western blot. (A) Immunoblot of SPARC and HRG proteins in PRE-CSF and POST-CSF samples. Fifty micrograms of PRE-CSF (lanes 1–3) or POST-CSF (lanes 4–6) CSF were subjected to the lanes. (B) and (C) The relative quantification of SPARC and HRG proteins from panels (A) using Image J software, respectively. Supplementary Table S1: an average of 428 unique proteins of all PRE-CSF and POST-CSF samples was identified by measuring peptides signal intensity for protein abundance.
