# Supplementary material for: Altered CSF Proteomic Profiling of Paediatric Acute Lymphocytic Leukemia Patients with CNS Infiltration
Source: J Oncol. 2019 May 2;2019:3283629. doi: 10.1155/2019/3283629 (PMC6521476; doi:10.1155/2019/3283629)
Supplement: Supplementary Materials — Supplementary Figure S1: Gene Ontology (GO) analysis of all identified proteins expressed in POST-CSF samples. Supplementary Figure S2: validation of SPARC and HRG protein peaks by western blot. (A) Immunoblot of SPARC and HRG proteins in PRE-CSF and POST-CSF samples. Fifty micrograms of PRE-CSF (lanes 1–3) or POST-CSF (lanes 4–6) CSF were subjected to the lanes. (B) and (C) The relative quantification of SPARC and HRG proteins from panels (A) using Image J software, respectively. Supplementary Table S1: an average of 428 unique proteins of all PRE-CSF and POST-CSF samples was identified by measuring peptides signal intensity for protein abundance. [file 3283629.f1.pdf]

Supplementary Table S1 An average of 428 unique proteins of all PRE-CSF and POST-CSF samples were identified by measuring peptides signal intensity for protein abundance.

Supplementary Figure S1 Gene Ontology (GO) analysis of all identified proteins that expressed in POST-CSF samples.

Supplementary Figure S2 Validation of SPARC and HRG protein peaks by western blot. (A) Immunoblot of SPARC and HRG proteins in PRE-CSF and POST-CSF samples. Fifty micrograms of PRE-CSF (lanes 1–3) or POST-CSF (lanes 4–6) CSF were subjected to the lanes. (B) and (C) The relative quantification of SPARC and HRG proteins from panels(A) using Image J software, respectively.

Supplementary Figure S1

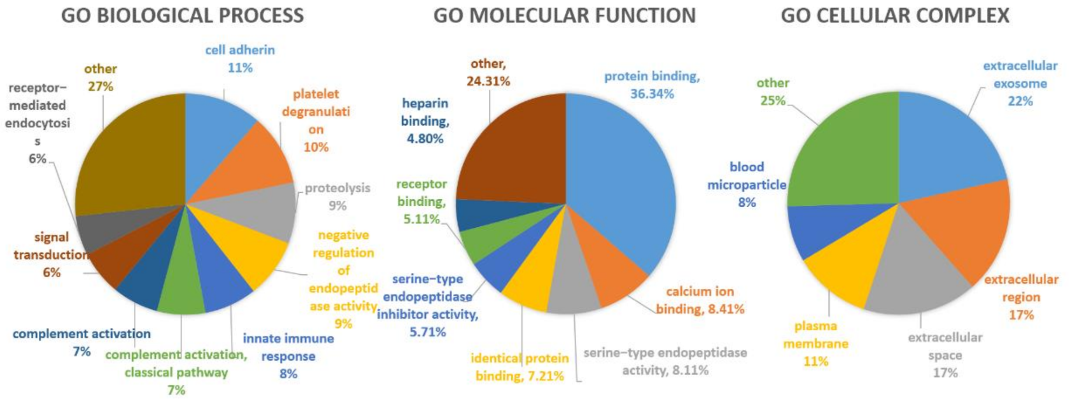

Supplementary Figure S2

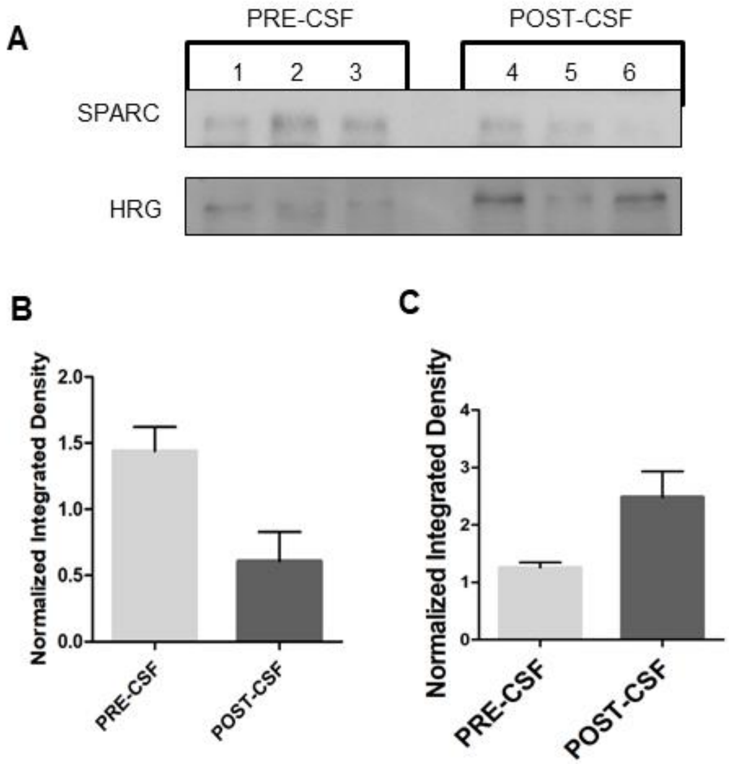

Supplementary Table S1

Protein IDs		Mol. weight [kDa]
P01031	complement C5(C5)	188.3
P55286	cadherin 8(CDH8)	88.253
P01613	immunoglobulin kappa variable 1D-33(IGKV1D-33)	12.848
O43493	trans-golgi network protein 2(TGOLN2)	51.019
Q9UBQ6	exostosin like glycosyltransferase 2(EXTL2)	37.466
Q92911	solute carrier family 5 member 5(SLC5A5)	68.666
P01702	immunoglobulin lambda variable 1-51(IGLV1-51)	12.249
Q16653	myelin oligodendrocyte glycoprotein(MOG)	28.193
Q99946	proline rich transmembrane protein 1(PRRT1)	31.43
Q7Z5M8	abhydrolase domain containing 12B(ABHD12B)	40.776
Q9UBX1	cathepsin F(CTSF)	53.366
P60033	CD81 molecule(CD81)	25.809
O75787	ATPase H+ transporting accessory protein 2(ATP6AP2)	39.008
Q9P232	contactin 3(CNTN3)	112.883
Q6EMK4	vasorin(VASN)	71.712
P01714	immunoglobulin lambda variable 3-19(IGLV3-19)	12.042
Q9NYX4	calcyon neuron specific vesicular protein(CALY)	23.434
P07357	complement C8 alpha chain(C8A)	65.163
P49641	mannosidase alpha class 2A member 2(MAN2A2)	130.539
P11047	laminin subunit gamma 1(LAMC1)	177.603
Q13555	calcium/calmodulin dependent protein kinase II gamma(CAMK2G)	62.607
Q12907	lectin, mannose binding 2(LMAN2)	40.229
P62328	thymosin beta 4, X-linked(TMSB4X)	5.053
Q6UX73	chromosome 16 open reading frame 89(C16orf89)	45.391

Q6UXD5	seizure related 6 homolog like 2(SEZ6L2)	97.56
P02654	apolipoprotein C1(APOC1)	9.3318
P04179	superoxide dismutase 2, mitochondrial(SOD2)	24.722
O94760	dimethylarginine dimethylaminohydrolase 1(DDAH1)	31.122
Q13519	prepronociceptin(PNOC)	20.295
P34096	ribonuclease A family member 4(RNASE4)	16.84
O95967	EGF containing fibulin like extracellular matrix protein 2(EFEMP2)	49.405
P01703	immunoglobulin lambda variable 1-40(IGLV1-40)	10.904
Q03591	complement factor H related 1(CFHR1)	37.651
P13473	lysosomal associated membrane protein 2(LAMP2)	44.961
P01621	immunoglobulin kappa variable 3-20(IGKV3-20)	10.729
P40925	malate dehydrogenase 1(MDH1)	36.426
P07585	decorin(DCN)	39.747
P23468	protein tyrosine phosphatase, receptor type D(PTPRD)	214.76
P01772	immunoglobulin heavy variable 3-33(IGHV3-33)	13.074
Q9NQX5	neural proliferation, differentiation and control 1(NPDC1)	34.516
P35908	keratin 2(KRT2)	65.865
Q5BIV9	shadow of prion protein homolog (zebrafish)(SPRN)	14.522
Q8N2C7	unc-80 homolog, NALCN activator(UNC80)	363.39
Q99969	retinoic acid receptor responder 2(RARRES2)	18.618
O60883	G protein-coupled receptor 37 like 1(GPR37L1)	52.77
Q9H0J4	glutamine rich 2(QRICH2)	180.827
P01127	platelet derived growth factor subunit B(PDGFB)	27.283
O95155	ubiquitination factor E4B(UBE4B)	146.185
P01605	immunoglobulin kappa variable 1D-33(IGKV1D-33)	12.848
P04156	prion protein(PRNP)	27.661

P09382	galectin 1(LGALS1)	14.716
Q9BYH1	seizure related 6 homolog like(SEZ6L)	111.782
P43251	biotinidase(BTD)	61.132
Q16620	neurotrophic receptor tyrosine kinase 2(NTRK2)	91.998
O95196	chondroitin sulfate proteoglycan 5(CSPG5)	60.016
Q86Y38	xylosyltransferase 1(XYLT1)	107.569
Q9BY67	cell adhesion molecule 1(CADM1)	48.509
Q5VYK3	KIAA0368(KIAA0368)	204.29
Q8IUB2	WAP four-disulfide core domain 3(WFDC3)	24.687
O94772	lymphocyte antigen 6 complex, locus H(LY6H)	14.669
Q9UPY3	dicer 1, ribonuclease III(DICER1)	218.682
A6NML5	transmembrane protein 212(TMEM212)	21.425
P18428	lipopolysaccharide binding protein(LBP)	53.384
Q6P387	chromosome 16 open reading frame 46(C16orf46)	43.417
Q15256	protein tyrosine phosphatase, receptor type R(PTPRR)	73.834
Q8N2S1	latent transforming growth factor beta binding protein 4(LTBP4)	173.435
Q9HAT2	sialic acid acetylesterase(SIAE)	58.315
P21926	CD9 molecule(CD9)	25.416
P01213	prodynorphin(PDYN)	28.385
P48061	C-X-C motif chemokine ligand 12(CXCL12)	10.666
P07711	cathepsin L(CTSL)	37.564
P21810	biglycan(BGN)	41.654
Q9BU40	chordin like 1(CHRDL1)	51.168
P02745	complement C1q A chain(C1QA)	26.017
A6NLU5	V-set and transmembrane domain containing 2B(VSTM2B)	30.297
Q15582	transforming growth factor beta induced(TGFBI)	74.681

Q9NXZ2	DEAD-box helicase 43(DDX43)	72.844
Q86XK3	SWI5 dependent homologous recombination repair protein 1(SFR1)	28.262
Q06828	fibromodulin(FMOD)	43.179
P04066	fucosidase, alpha-L- 1, tissue(FUCA1)	53.619
P04003	complement component 4 binding protein alpha(C4BPA)	67.033
Q6ZWL3	cytochrome P450 family 4 subfamily V member 2(CYP4V2)	60.723
O14498	immunoglobulin superfamily containing leucine rich repeat(ISLR)	45.997
Q5FWE3	proline rich transmembrane protein 3(PRRT3)	102.197
P23582	natriuretic peptide C(NPPC)	13.246
Q9Y5Y7	lymphatic vessel endothelial hyaluronan receptor 1(LYVE1)	35.213
Q16769	glutaminyl-peptide cyclotransferase(QPCT)	40.877
Q9UNN8	protein C receptor(PROCR)	26.671
Q9HCB6	spondin 1(SPON1)	90.973
P14543	nidogen 1(NID1)	136.377
P16070	CD44 molecule (Indian blood group)(CD44)	81.538
Q14508	WAP four-disulfide core domain 2(WFDC2)	12.993
Q96PD5	peptidoglycan recognition protein 2(PGLYRP2)	62.216
P29966	myristoylated alanine rich protein kinase C substrate(MARCKS)	31.555
Q92820	gamma-glutamyl hydrolase(GGH)	35.964
Q9NZT1	calmodulin like 5(CALML5)	15.892
Q9UBX7	kallikrein related peptidase 11(KLK11)	31.059

P27169	paraoxonase 1(PON1)	39.731
P01602	immunoglobulin kappa variable 1-5(IGKV1-5)	12.768
P05109	S100 calcium binding protein A8(S100A8)	10.834
Q9H3G5	carboxypeptidase, vitellogenic like(CPVL)	54.164
Q9Y287	integral membrane protein 2B(ITM2B)	30.338
P19827	inter-alpha-trypsin inhibitor heavy chain 1(ITIH1)	101.389
P01040	cystatin A(CSTA)	11.006
P80748	immunoglobulin lambda variable 3-21(IGLV3-21)	12.446
Q96RL7	vacuolar protein sorting 13 homolog A(VPS13A)	360.276
P01765	immunoglobulin heavy variable 3-23(IGHV3-23)	11.612
P55083	microfibrillar associated protein 4(MFAP4)	28.648
Q9UHL4	dipeptidyl peptidase 7(DPP7)	54.341
P29622	serpin family A member 4(SERPINA4)	48.542
Q14C87	transmembrane protein 132D(TMEM132D)	122.309
Q6MZW2	follistatin like 4(FSTL4)	93.096
O75144	inducible T-cell costimulator ligand(ICOSLG)	33.349
Q86UX2	inter-alpha-trypsin inhibitor heavy chain family member 5(ITIH5)	104.576
P08174	CD55 molecule (Cromer blood group)(CD55)	41.4
P47972	neuronal pentraxin 2(NPTX2)	47.042
P02792	ferritin light chain(FTL)	20.02
Q9NT99	leucine rich repeat containing 4B(LRRC4B)	76.434
Q9UQM7	calcium/calmodulin dependent protein kinase II alpha(CAMK2A)	54.088
Q92520	family with sequence similarity 3 member C(FAM3C)	24.68
P01603	immunoglobulin kappa variable 1D-33(IGKV1D-33)	12.848
P62826	RAN, member RAS oncogene family(RAN)	24.423

P00491	purine nucleoside phosphorylase(PNP)	32.118
P06702	S100 calcium binding protein A9(S100A9)	13.242
P00918	carbonic anhydrase 2(CA2)	29.246
P04040	catalase(CAT)	59.756
P32119	peroxiredoxin 2(PRDX2)	21.892
P02042	hemoglobin subunit delta(HBD)	16.055
P69892	hemoglobin subunit gamma 2(HBG2)	16.126
P13598	intercellular adhesion molecule 2(ICAM2)	30.654
P35555	fibrillin 1(FBN1)	312.237
Q86UD1	out at first homolog(OAF)	30.688
P55283	cadherin 4(CDH4)	100.281
Q9HDB5	neurexin 3(NRXN3)	69.305
P23528	cofilin 1(CFL1)	18.502
Q8TB22	spermatogenesis associated 20(SPATA20)	87.899
Q9UN36	NDRG family member 2(NDRG2)	40.798
P08637	Fc fragment of IgG receptor IIIa(FCGR3A)	29.089
P09104	enolase 2(ENO2)	47.269
Q9UMF0	intercellular adhesion molecule 5(ICAM5)	97.116
Q96B86	repulsive guidance molecule family member a(RGMA)	49.347
P48745	nephroblastoma overexpressed(NOV)	39.162
P06733	enolase 1(ENO1)	47.169
Q6NW4 0	repulsive guidance molecule family member b(RGMB)	47.547
Q96PX8	SLIT and NTRK like family member 1(SLITRK1)	77.735
P01717	immunoglobulin lambda variable 3-25(IGLV3-25)	12.011
Q13835	plakophilin 1(PKP1)	82.861
P32004	L1 cell adhesion molecule(L1CAM)	140.003
Q99674	cell growth regulator with EF-hand domain 1(CGREF1)	31.905

O00468	agrin(AGRN)	217.32
Q01469	fatty acid binding protein 5(FABP5)	15.164
Q08554	desmocollin 1(DSC1)	99.987
P30530	AXL receptor tyrosine kinase(AXL)	98.337
P35326	small proline rich protein 2A(SPRR2A)	7.965
Q9BVG8	kinesin family member C3(KIFC3)	92.775
Q02413	desmoglein 1(DSG1)	113.748
P14923	junction plakoglobin(JUP)	81.744
Q6UX71	plexin domain containing 2(PLXDC2)	59.583
O15195	villin like(VILL)	95.907
O00451	GDNF family receptor alpha 2(GFRA2)	51.544
Q5SRE5	nucleoporin 188(NUP188)	196.043
P00915	carbonic anhydrase 1(CA1)	28.87
P00748	coagulation factor XII(F12)	67.791
P08185	serpin family A member 6(SERPINA6)	45.14
P11021	heat shock protein family A (Hsp70) member 5(HSPA5)	72.332
P02144	myoglobin(MB)	17.184
O15354	G protein-coupled receptor 37(GPR37)	67.114
P07451	carbonic anhydrase 3(CA3)	29.557
P69905	hemoglobin subunit alpha 1(HBA1)	15.257
P68871	hemoglobin subunit beta(HBB)	15.998
Q6UWP8	suprabasin(SBSN)	60.541
P49748	acyl-CoA dehydrogenase, very long chain(ACADVL)	70.39
P25705	ATP synthase, H+ transporting, mitochondrial F1 complex, alpha subunit 1, cardiac muscle(ATP5A1)	59.751
Q06481	amyloid beta precursor like protein 2(APLP2)	86.956
Q9BQT9	calsyntenin 3(CLSTN3)	106.098

P49908	selenoprotein P(SELENOP)	43.174
P12235	solute carrier family 25 member 4(SLC25A4)	33.064
Q9UJJ9	N-acetylglucosamine-1-phosphate transferase gamma subunit(GNPTG)	33.974
Q6UW01	cerebellin 3 precursor(CBLN3)	21.521
Q53EL9	seizure related 6 homolog(SEZ6)	107.425
O00391	quiescin sulfhydryl oxidase 1(QSOX1)	82.578
P43121	melanoma cell adhesion molecule(MCAM)	71.607
Q96JF0	ST6 beta-galactoside alpha-2,6-sialyltransferase 2(ST6GAL2)	60.158
P09603	colony stimulating factor 1(CSF1)	60.179
O00584	ribonuclease T2(RNASET2)	29.481
P07333	colony stimulating factor 1 receptor(CSF1R)	107.98
P63104	tyrosine 3-monooxygenase/tryptophan 5-monooxygenase activation protein zeta(YWHAZ)	27.745
P51884	lumican(LUM)	38.429
Q9UBX5	fibulin 5(FBLN5)	50.18
P62158	calmodulin 1(CALM1)	16.837
P04075	aldolase, fructose-bisphosphate A(ALDOA)	39.42
Q92932	protein tyrosine phosphatase, receptor type N2(PTPRN2)	111.271
P80723	brain abundant membrane attached signal protein 1(BASP1)	22.693
P61916	melanoma cell adhesion molecule(MCAM)	16.57
Q9UM73	anaplastic lymphoma receptor tyrosine kinase(ALK)	176.44
P05156	complement factor I(CFI)	65.75
P04196	histidine rich glycoprotein(HRG)	59.578

B9A064	immunoglobulin lambda like polypeptide 5(IGLL5)	23.063
Q9UI43	mitochondrial rRNA methyltransferase 2(MRM2)	27.424
P01781	immunoglobulin heavy variable 3-7(IGHV3-7)	12.943
O60241	adhesion G protein-coupled receptor B2(ADGRB2)	172.656
O75882	attractin(ATRN)	158.537
O60216	RAD21 cohesin complex component(RAD21)	71.69
P23470	protein tyrosine phosphatase, receptor type G(PTPRG)	162.003
P06310	immunoglobulin kappa variable 2-30(IGKV2-30)	13.185
Q9BZR6	reticulon 4 receptor(RTN4R)	50.708
P02656	apolipoprotein C3(APOC3)	10.852
P01625	immunoglobulin kappa variable 4-1(IGKV4-1)	13.38
Q7Z3B1	neuronal growth regulator 1(NEGR1)	38.719
Q7Z7M0	multiple EGF like domains 8(MEGF8)	303.1
P01610	immunoglobulin kappa variable 1-17(IGKV1-17)	12.779
O60888	cutA divalent cation tolerance homolog(CUTA)	19.116
Q13449	limbic system-associated membrane protein(LSAMP)	37.393
Q96FE7	phosphoinositide-3-kinase interacting protein 1(PIK3IP1)	28.248
P01861	immunoglobulin heavy constant gamma 4 (G4m marker)(IGHG4)	35.94
P01593	immunoglobulin kappa variable 1D-33(IGKV1D-33)	12.848
Q8IWB9	testis expressed 2(TEX2)	125.3
P06703	S100 calcium binding protein A6(S100A6)	10.18
P35580	myosin heavy chain 10(MYH10)	228.999
Q9NYL9	tropomodulin 3(TMOD3)	39.595
P35749	myosin heavy chain 11(MYH11)	227.339
Q99878	histone cluster 1 H2A family member j(HIST1H2AJ)	13.936
Q13813	spectrin alpha, non-erythrocytic 1(SPTAN1)	284.593

Q9Y2T3	guanine deaminase(GDA)	51.003
P17677	growth associated protein 43(GAP43)	24.803
Q9NZ53	podocalyxin like 2(PODXL2)	65.076
P06681	complement C2(C2)	83.267
O00159	myosin IC(MYO1C)	121.682
Q6UXB8	peptidase inhibitor 16(PI16)	49.471
Q13228	selenium binding protein 1(SELENBP1)	52.391
O75326	semaphorin 7A (John Milton Hagen blood group)(SEMA7A)	74.824
O14773	tripeptidyl peptidase 1(TPP1)	61.248
P13611	versican(VCAN)	372.82
Q9Y646	carboxypeptidase Q(CPQ)	51.888
Q13508	ADP-ribosyltransferase 3(ART3)	43.923
P14151	selectin L(SELL)	42.187
P15924	desmoplakin(DSP)	331.77
Q08629	SPARC/osteonectin, cwcv and kazal like domains proteoglycan 1(SPOCK1)	49.124
Q24JP5	transmembrane protein 132A(TMEM132A)	110.11
P09972	aldolase, fructose-bisphosphate C(ALDOC)	39.455
P01871	immunoglobulin heavy constant mu(IGHM)	49.44
P00746	complement factor D(CFD)	27.033
O94769	extracellular matrix protein 2(ECM2)	79.789
Q8IWU5	sulfatase 2(SULF2)	100.455
Q8TAG5	V-set and transmembrane domain containing 2A(VSTM2A)	25.833
Q969P0	immunoglobulin superfamily member 8(IGSF8)	65.033
Q96S96	phosphatidylethanolamine binding protein 4(PEBP4)	25.733
P04180	lecithin-cholesterol acyltransferase(LCAT)	49.577

P42785	prolylcarboxypeptidase(PRCP)	55.8
P78324	signal regulatory protein alpha(SIRPA)	54.967
Q92563	SPARC/osteonectin, cwcv and kazal like domains proteoglycan 2(SPOCK2)	46.779
Q12841	follistatin like 1(FSTL1)	34.986
Q86VB7	CD163 molecule(CD163)	125.451
Q02818	nucleobindin 1(NUCB1)	53.879
P04208	immunoglobulin lambda variable 1-47(IGLV1-47)	11.725
P08123	collagen type I alpha 2 chain(COL1A2)	129.314
Q14982	opioid binding protein/cell adhesion molecule like(OPCML)	38.007
O94919	endonuclease domain containing 1(ENDOD1)	55.017
P17936	insulin like growth factor binding protein 3(IGFBP3)	31.674
Q9Y6R7	Fc fragment of IgG binding protein(FCGBP)	572.01
P60174	triosephosphate isomerase 1(TPI1)	30.791
Q13740	activated leukocyte cell adhesion molecule(ALCAM)	65.102
O15394	neural cell adhesion molecule 2(NCAM2)	93.045
P39060	collagen type XVIII alpha 1 chain(COL18A1)	178.19
Q8TCZ2	CD99 molecule like 2(CD99L2)	27.986
P23515	oligodendrocyte myelin glycoprotein(OMG)	49.608
P18135	immunoglobulin kappa variable 3-20(IGKV3-20)	14.073
O94910	adhesion G protein-coupled receptor L1(ADGRL1)	162.717
Q9Y4C0	neurexin 3(NRXN3)	180.599
Q9P2S2	neurexin 2(NRXN2)	184.98
Q8NFZ8	cell adhesion molecule 4(CADM4)	42.785
P04406	glyceraldehyde-3-phosphate dehydrogenase(GAPDH)	36.053
P43652	afamin(AFM)	69.068
O94856	neurofascin(NFASC)	150.02

Q13332	protein tyrosine phosphatase, receptor type S(PTPRS)	217.04
P35579	myosin heavy chain 9(MYH9)	226.532
Q9UPU3	sortilin related VPS10 domain containing receptor 3(SORCS3)	135.755
P01344	insulin like growth factor 2(IGF2)	20.14
P02675	fibrinogen beta chain(FGB)	55.928
Q8NBJ4	golgi membrane protein 1(GOLM1)	45.333
Q16610	extracellular matrix protein 1(ECM1)	60.673
Q5T749	keratinocyte proline rich protein(KPRP)	64.135
Q92859	neogenin 1(NEO1)	160.017
P02679	fibrinogen gamma chain(FGG)	51.511
P55058	phospholipid transfer protein(PLTP)	54.739
Q5BLP8	chromosome 4 open reading frame 48(C4orf48)	10.17
Q14624	inter-alpha-trypsin inhibitor heavy chain family member 4(ITIH4)	103.36
P08253	matrix metallopeptidase 2(MMP2)	73.882
P0C0L5	complement C4B (Chido blood group)(C4B)	192.75
P19021	peptidylglycine alpha-amidating monooxygenase(PAM)	108.33
P36222	chitinase 3 like 1(CHI3L1)	42.625
P16035	TIMP metallopeptidase inhibitor 2(TIMP2)	24.399
Q8N126	cell adhesion molecule 3(CADM3)	43.299
P17174	glutamic-oxaloacetic transaminase 1(GOT1)	46.247
P22692	insulin like growth factor binding protein 4(IGFBP4)	27.934
Q99574	serpin family I member 1(SERPINI1)	46.427
Q9P121	neurotrimin(NTM)	37.971
P23471	protein tyrosine phosphatase, receptor type Z1(PTPRZ1)	254.58
P01033	TIMP metallopeptidase inhibitor 1(TIMP1)	23.171

P12109	collagen type VI alpha 1 chain(COL6A1)	108.53
Q02246	contactin 2(CNTN2)	113.39
P12259	coagulation factor V(F5)	251.703
P07195	lactate dehydrogenase B(LDHB)	36.638
Q15904	ATPase H+ transporting accessory protein 1(ATP6AP1)	52.026
Q96GW 7	brevican(BCAN)	99.117
P54764	EPH receptor A4(EPHA4)	109.86
Q15113	procollagen C-endopeptidase enhancer(PCOLCE)	47.972
P78509	reelin(RELN)	388.38
P22352	glutathione peroxidase 3(GPX3)	25.552
P14618	pyruvate kinase, muscle(PKM)	57.936
P02760	alpha-1-microglobulin/bikunin precursor(AMBP)	38.999
P63261	actin gamma 1(ACTG1)	41.792
P0DJI8	serum amyloid A1(SAA1)	13.532
P02753	retinol binding protein 4(RBP4)	23.01
P54289	calcium voltage-gated channel auxiliary subunit alpha2delta 1(CACNA2D1)	124.568
P01766	immunoglobulin heavy variable 3-13(IGHV3-13)	13.227
Q8TEU8	WAP, follistatin/kazal, immunoglobulin, kunitz and netrin domain containing 2(WFIKKN2)	63.941
P08697	serpin family F member 2(SERPINF2)	54.566
P02452	collagen type I alpha 1 chain(COL1A1)	138.941
P05408	secretogranin V(SCG5)	23.729
P10643	complement C7(C7)	93.517
Q15818	neuronal pentraxin 1(NPTX1)	47.122
P07858	cathepsin B(CTSB)	37.822

P05546	serpin family D member 1(SERPIND1)	57.07
P02765	alpha 2-HS glycoprotein(AHSG)	39.324
P16870	carboxypeptidase E(CPE)	53.15
P02748	complement C9(C9)	63.173
P02746	complement C1q B chain(C1QB)	26.722
P24592	insulin like growth factor binding protein 6(IGFBP6)	25.322
Q96KN2	carnosine dipeptidase 1(CNDP1)	56.705
P19022	cadherin 2(CDH2)	99.808
O43505	beta-1,4-glucuronyltransferase 1(B4GAT1)	47.119
P02747	complement C1q C chain(C1QC)	25.773
Q14118	dystroglycan 1(DAG1)	97.44
P01617	immunoglobulin kappa variable 2D-28(IGKV2D-28)	12.957
P17900	GM2 ganglioside activator(GM2A)	20.838
O14594	neurocan(NCAN)	143.09
Q9ULB1	neurexin 1(NRXN1)	161.88
P00736	complement C1r(C1R)	80.118
P04433	immunoglobulin kappa variable 3D-11(IGKV3D-11)	12.575
P00441	superoxide dismutase 1, soluble(SOD1)	15.936
P07998	ribonuclease A family member 1, pancreatic(RNASE1)	17.644
P07339	cathepsin D(CTSD)	44.552
P04004	vitronectin(VTN)	54.305
P18065	insulin like growth factor binding protein 2(IGFBP2)	34.814
P55290	cadherin 13(CDH13)	78.286
P09871	complement C1s(C1S)	76.684
P08571	CD14 molecule(CD14)	40.076
P30086	phosphatidylethanolamine binding protein 1(PEBP1)	21.057
P02652	apolipoprotein A2(APOA2)	11.175
P02750	leucine rich alpha-2-glycoprotein 1(LRG1)	38.178

Q9NQ79	cartilage acidic protein 1(CRTAC1)	71.42
P01860	immunoglobulin heavy constant gamma 3 (G3m marker)(IGHG3)	41.287
P00734	coagulation factor II, thrombin(F2)	70.036
P07225	protein S (alpha)(PROS1)	75.122
P20774	osteoglycin(OGN)	33.922
P04216	Thy-1 cell surface antigen(THY1)	17.935
P62987	ubiquitin A-52 residue ribosomal protein fusion product 1(UBA52)	14.728
O95502	neuronal pentraxin receptor(NPTXR)	52.846
P01042	kininogen 1(KNG1)	71.957
P13591	neural cell adhesion molecule 1(NCAM1)	94.573
Q66K66	transmembrane protein 198(TMEM198)	39.474
Q12860	contactin 1(CNTN1)	113.32
P61769	beta-2-microglobulin(B2M)	13.714
P13521	secretogranin II(SCG2)	70.94
P01008	serpin family C member 1(SERPINC1)	52.602
P25311	alpha-2-glycoprotein 1, zinc-binding(AZGP1)	34.258
P00747	plasminogen(PLG)	90.568
P08294	superoxide dismutase 3, extracellular(SOD3)	25.851
P09486	secreted protein acidic and cysteine rich(SPARC)	34.632
P01210	proenkephalin(PENK)	30.787
P01859	immunoglobulin heavy constant gamma 2 (G2m marker)(IGHG2)	35.9
P04217	alpha-1-B glycoprotein(A1BG)	54.253
P13987	CD59 molecule(CD59)	14.177
P01623	immunoglobulin kappa variable 3-20(IGKV3-20)	11.746
P01876	immunoglobulin heavy constant alpha 1(IGHA1)	37.654

Q99435	neural EGFL like 2(NELL2)	91.346
Q92876	kallikrein related peptidase 6(KLK6)	26.855
P05067	amyloid beta precursor protein(APP)	86.942
P0CG05	immunoglobulin lambda constant 2(IGLC2)	11.293
P05452	C-type lectin domain family 3 member B(CLEC3B)	22.537
Q12805	EGF containing fibulin like extracellular matrix protein 1(EFEMP1)	54.64
P02749	apolipoprotein H(APOH)	38.298
Q08380	galectin 3 binding protein(LGALS3BP)	65.33
O15240	VGF nerve growth factor inducible(VGF)	67.257
O00533	cell adhesion molecule L1 like(CHL1)	135.07
P01834	immunoglobulin kappa constant(IGKC)	11.609
Q9UHG2	proprotein convertase subtilisin/kexin type 1 inhibitor(PCSK1N)	27.372
P07602	prosaposin(PSAP)	58.112
P05155	serpin family G member 1(SERPING1)	55.154
P06727	apolipoprotein A4(APOA4)	45.398
P08603	complement factor H(CFH)	139.09
P00751	complement factor B(CFB)	85.532
P51693	amyloid beta precursor like protein 1(APLP1)	72.176
O94985	calsyntenin 1(CLSTN1)	109.79
Q14515	SPARC like 1(SPARCL1)	75.207
Q16270	insulin like growth factor binding protein 7(IGFBP7)	29.13
Q8WXD2	secretogranin III(SCG3)	53.005
P10645	chromogranin A(CHGA)	50.688
Q92823	neuronal cell adhesion molecule(NRCAM)	143.89
P01011	serpin family A member 3(SERPINA3)	47.65

P23142	fibulin 1(FBLN1)	77.213
P02751	fibronectin 1(FN1)	262.62
P19652	orosomucoid 2(ORM2)	23.602
P02774	GC, vitamin D binding protein(GC)	52.963
P10451	secreted phosphoprotein 1(SPP1)	35.422
P00450	ceruloplasmin(CP)	122.2
P01023	alpha-2-macroglobulin(A2M)	163.29
Q9UBP4	dickkopf WNT signaling pathway inhibitor 3(DKK3)	38.39
P01019	angiotensinogen(AGT)	53.154
P02647	apolipoprotein A1(APOA1)	30.777
P01009	serpin family A member 1(SERPINA1)	46.736
Q13822	ectonucleotide pyrophosphatase/phosphodiesterase 2(ENPP2)	98.993
P05060	chromogranin B(CHGB)	78.275
P05090	apolipoprotein D(APOD)	21.275
P0C0L4	complement C4A (Rodgers blood group)(C4A)	192.78
P01034	cystatin C(CST3)	15.799
P00738	haptoglobin(HP)	45.205
P01024	complement C3(C3)	187.15
P02763	orosomucoid 1(ORM1)	23.511
P36955	serpin family F member 1(SERPINF1)	46.312
P10909	clusterin(CLU)	52.494
P41222	prostaglandin D2 synthase(PTGDS)	21.029
P01857	immunoglobulin heavy constant gamma 1 (G1m marker)(IGHG1)	36.105
P02790	hemopexin(HPX)	51.676
P02649	apolipoprotein E(APOE)	36.154
P02787	transferrin(TF)	77.063

P02766	transthyretin(TTR)	15.887
